# Research on antidepressants in India

**DOI:** 10.4103/0019-5545.69263

**Published:** 2010-01

**Authors:** Ajit Avasthi, Sandeep Grover, Munish Aggarwal

**Affiliations:** Department of Psychiatry, Postgraduate Institute of Medical Education and Research, Chandigarh, India

**Keywords:** Antidepressants, research, India

## Abstract

Data suggests that antidepressants are useful in the management of depressive disorders, anxiety disorders, sexual dysfunction, eating disorders, impulse control disorders, enuresis, aggression and some personality disorders. Research focusing on the usefulness of antidepressants in India has more or less followed the trends seen in the West. Most of the studies conducted in India have evaluated various antidepressants in depression. In this article, we review studies conducted in India on various antidepressants. The data suggests that antidepressants have been evaluated mainly in the acute phase treatment and rare studies have evaluated the efficacy in continuation phase treatment.

## INTRODUCTION

Antidepressants as a class of drugs are used primarily in the management of depressive disorders and anxiety disorders. However, this class of drugs is also used for the management of sexual dysfunction, eating disorders, impulse control disorders, enuresis, aggression and some personality disorders.

Over the years many classes of antidepressants have become available in India, some of which have stood the test of time and are still in use and some, which are no more marketed or are no more a favorite of clinicians. The research focusing the usefulness of antidepressants in India has more or less followed the trends in the west; however, some of the antidepressants drugs which have been marketed have not been evaluated as thoroughly as others.

Most of the studies done in India have evaluated various antidepressants in depression. There are very few studies which have evaluated antidepressants in conditions other than depressive disorders. In this article, we review studies done in India on various antidepressants. The review shall focus on the research published in Indian Journal of Psychiatry and studies reported in PubMed indexed journals on efficacy, effectiveness, usefulness and tolerability issues of antidepressants in human subjects.

### Efficacy/effectiveness in Depression

The trials done to evaluate the efficacy of antidepressants can be divided into studies evaluating an antidepressant (no comparator studies), efficacy of an antidepressant with placebo as a comparator, comparing efficacy of 2 active drugs and those evaluating the efficacy of antidepressants with other modalities of treatment like electro-convulsive therapy or psychological treatment.

### Non Comparative Studies

A total of 18 open trials without a comparator group have been conducted to evaluate the efficacy of various antidepressants [Table 1].[1–18] Studies done in the 1960s evaluated the efficacy of tricyclic antidepressants. Later studies have evaluated the efficacy of nitroxazepine, centpropazine, amineptine, tianeptine, sertraline and milnacipran. Studies done prior to 1990s have not used any standardized rating scales, most of these also did not mention the diagnostic criteria used for the diagnosis. These studies included subjects diagnosed with various subtypes like reactive depression, endogenous depression, psychoneurotic depression, melancholic depression etc. However, the studies done after 1990s have recruited subjects diagnosed on the basis of DSM or ICD-10 RDC criteria and used standardized rating scales to evaluate the efficacy or effectiveness. The sample sizes of the studies have varied a lot, most of the earlier studies included less than 50 subjects; however, some of the recent studies have included more than 300 subjects. Most of these trials have evaluated the outcome after six weeks. All these trials have shown that various tricyclic antidepressants, nitroxazepine, centpropazine, amineptine, tianeptine, sertraline, escitalopram and milnacipran are efficacious in treatment of depression. The trial which evaluated the efficacy of sertraline also showed that treatment of depression with sertraline leads to reduction in cardiac events post myocardial infarction.[13] A recently published trial, which evaluated the efficacy of milnacipran, included subjects who had suffered from stroke.[18] It is also one of the few trials which have included subjects more than 65 years of age. The trial done by Margoob *et al*.[17] in addition to the efficacy of escitalopram, have also shown that gene polymorphism plays an important role in the treatment response to various antidepressants.

**Table 1:** Non comparative studies evaluating efficacy of antidepressants in depressive disorders

Authors	Duration (in weeks)	Sample size/scale/design	Medication(s)	Dose(s) in mg	Outcome	Side-effects
Dube and Narendra[1]	Variable	N = 11	IMN	75-225	Subjects with endogenous depression showed marked improvement or complete recovery, the psychoneurotic variety showed little and no improvement was seen in schiz group	Increase in pulse rate, dryness of mouth, hypotension, dilatation of pupils, stomatitis, constipation, excitement and sinking sensation
Davis[2]	3-till needed	N = 30	AMT	75-150	Sixteen subjects became Sx free; 8 subjects were substantially improved; taken together-77% improved, 3 subjects relapsed, when treatment was discontinued	Dermatitis, constipation, restlessness and panic, dry month
Kishore and Murti Rao[3]	3-6 months	N = 10	AMT	75-150	Two lost on follow-up One patient improved slightly, 5-considerably improved, 1 -completely free of depression, 1- did not improve (hypochondriasis with sx of depression)	One patient- severe generalized Pruritus. One patient-dizziness, ataxia, drowsiness, blurring of vision, dryness of mouth, nausea, sweating and pains in the knee joints. One patient- took overdose
Master,[4]	4	N = 20	PTP	20-60	Effective in treatment of depression	Dryness of the month, mild drowsiness, dizziness, headache, asthenia
Kishore and Sharma[5]	15	N = 16	TIMN	25-300	8/12 subjects who completed the trial showed response. Superior results were observed in endogenous depression and there was little or no effect on reactive neurotic depression Tension, restlessness, agitation, suicidal tendencies, anxiety, insomnia and somatic complaints were relieved earlier than the depression.	Dryness of the month, mild drowsiness, dizziness, headache, asthenia
Sharma *et al*.[6]	10	N = 78	D-IMN	75-300	D-IMN was found to be effective Remission rates highest in the retarded and reactive depression; lowest in agitated depression	Dryness of mouth, palpitation, agitation, giddiness, constipation S/E were of milder severity
Shah *et al*.[7]	6	N = 104	IMN + PRZ	75-225 75-225	Compound proved to be effective in controlling the depression with anxiety	Commonly reported S/E included dry mouth, tremors, giddiness, constipation, difficulty in visual accommodation
Boral and Shah[8]	6	N = 32; DSM-III, HDR	AMN	100-200	AMN was effective as soon as day 7 of treatment and this effect improved continuously throughout the study	Safe
Bhatt *et al*.[9]	6	N = 10; Pharmacokinetic study DSM-IIIR HDRS	NTZ	75-225	The overall reduction in HDRS score was about 50% by 6 weeks. The HDRS score showed a steady reduction between day 14 and 42 when the levels of NTZ and desmethyl metabolites were maintained between 176.5 ng/ml to 251 ng/ml The plasma levels of NTZ (ng/ml) showed a rise from a mean level of 47.0 ± 7.3 on day 1 (dose 75 mg) to 129.8 ± 24.6 on day 7 (dose 150 mg) (*P* < 0.01) and remained steady till day 21	No severe A/E reported
Srivastava *et al*.[10]	4	N = 42; HDRS, RDC criteria multicentric	CTZ	40-120	Overall 81 % of subjects responded Antidepressant effect was seen in 9 subjects after 1 week, in 28 subjects after 2 weeks and in all 34 subjects after 3 weeks of therapy	11 subjects experienced S/E in the form of giddiness, headache, dryness of mouth and weakness
Parikh *et al*.[11]	6	N = 49; DSM-IIIR; HDRS, HARS, MADRS Dysthymia/MDD	TPN	37.5-75	There was a significant drop in scores on the HDRS, HARS and MADRS by week 2 which was sustained till week 6 (*P* < 0.05)	Common S/E were nausea, giddiness and drowsiness.
Channabasavanna and Khanna[12]	6	N = 50; HDRS, MADRS, CGI; ZUNG	AMN	200	At the end of 6 weeks treatment there was significant reduction in HDRS	No effect of AMN on heart rate and blood pressure
Sonawalla *et al*.[13]	6	N = 314; multicentric study outcome measure: Dropout rates MADRS, HARS	TPN	37.5	Intention to treat analysis showed that 7 subjects (2.3%) discontinued treatment due to S/Es Subjects with an improvement of at least 50% from baseline on MADRS- 53.0% at 6 week; at least 50% on HARS- 52.2% at 6 weeks	Well tolerated
Mohapatra *et al*.[14]	6 months	N = 17; Patient of AMI with MDD randomized, single blind	STN Vs Cardiac TAU	50-200	STN significantly better that TAU At the end of six months ten (90.9%) out of 11 subjects in intervention group achieved remission compared to two (33.3%) out of 3 of the TAU group Cardiac events were reported less in this group than those who continued only cardiac TAU	No dropouts due to non-response or S/E
Gada[15]	60 day	N = 320; MDD, HDRS	TPN	37.5	Mean HDRS score decreased from 10.9 ± 3.2 at baseline to 6.9 ± 2.7 at day 60 (*P* < 0.01), with more than half the subjects showing > 50% improvement in HDRS score. Mean compliance with the medication was 91%.	No patient withdrew due to S/E, which were reported in 23 subjects (7.2%).
Pinto *et al*.[16]	8	N = 119; MDD, DSM-IV, HDRS, MADRS	ESC	10-20	By week 8, 76.9% subjects had responded to treatment (≥ 50% reduction in MADRS total score).	ESC was well tolerated, and only 2 subjects (1.7%) withdrew from the study due to A/E. There were no serious A/Es
Margoob *et al*.[17]	6	N = 57; DSM-IV, HDRS	ESC	20	At the third week of treatment, 12 (63.15%) of Group ll (group without functionally dominant genes) had a 50% reduction HRDS score compared to only 4 (10.52%) of the s (group with functionally dominant genes) group (*P* < 0.001) Almost same treatment response trend continued up to the end of week	
Arora and Kaur[18]	6	N = 15; post stroke depression DSM-IV, HDRS	MSC	100-200	85.71% (12/14) of subjects completing the study were in remission	Not mentioned

IMN - Imipramine; schiz - Schizophrenia; AMT - Amitriptyline; PTP - Protriptyline; TIMN - Trimipramine; D-IMN - Desmethyl-Imipramine; S/E - Side-effects; PRZ - Promazine; HDRS - Hamilton depression rating scale; AMN - Amineptine; NTZ - Nitroxazepine; A/E - Adverse effect; RDC - Research Diagnostic Criteria; CTZ - Centpropazine; HARS - Hamilton Anxiety Rating Scale; MADRS - Montgomery Asberg Depression Rating Scale; MDD - Major Depressive Disorder; TPN - Tianeptine; CGI - Clinical Global Improvement Scale; ZUNG - Zung Depression Rating Scale; AMI - Acute myocardial infarction; STN - Sertraline; TAU - Cardiac treatment as usual; ESC - Escitalopram; MSC - Milnacipran

### Placebo-Controlled Trials

Six placebo controlled trials have evaluated the efficacy of tricyclic antidepressants in depression [Table 2].[19–24] Four of these trials have been double blind controlled trials,[19–22] one recruited subjects by consecutive sampling,[23] and one followed cross over design.[24] The duration of these trials has varied from four to eight weeks and these have included 16 to 96 subjects. Of the five trials, one evaluated the efficacy of imipramine in depressive symptoms in schizophrenia[24] and another included subjects with endogenous depression only.[23] Five of these trials showed that amitriptyline, imipramine, protriptyline and trimipramine are better than placebo in the management of depression;[19–23] however, the trial which evaluated the efficacy of imipramine for depressive symptoms in schizophrenia showed negative findings.[24]

**Table 2:** Placebo controlled trials evaluating the efficacy/effectiveness of antidepressants in depressive disorders

Authors	Duration (in weeks)	Sample size/scale/design	Medication(s)	Dose(s) in mg	Outcome	Side-effects
Master[19]	12	N=60 DBCT	AMT	75-150	Only 12 subjects completed the trial Subjects in the AMT group showed better response compared to placebo	Commonly reported side-effects; dryness of mouth, constipation
Shah *et al*.[20]	4	N=56 DBCT	AMT	75-150	62% of subjects in AMT group improved compared to 29% in the PLB group	S/E more frequently seen in AMT group: Giddiness, constipation, dryness of month, drowsiness, hypomania
Bassa and Vora[21]	4	N = 96; DBCT	IMN	150	60% of IMN subjects showed more than 50% response No difference between PLB and IMN	Not reported
Teja *et al*.[22]	8	N = 65; HDRS Crossover after 4 weeks	PTP	10-40	On drug treatment -29/46 subjects (63.0%) on PTP were better at 4 weeks with 19.5% rated as recovered, 21.5% rated as markedly improved, and 21.5% were rated as improved In the PLB arm only 2(10.5%) of the 19 subjects were rated as improved The differences between the drug and PLB groups were significant Drug was better than placebo in all types of depression More effective in psychotic depressives and involutional melancholia	S/E with PTP reported more frequently than that seen in placebo group: Dryness of mouth or bad taste, dizziness, increase thirst, constipation, blurring of vision, delayed micturition, epigastric or retrosternal distress, weakness or fatigue, itching, tremor
Nandi and Ajmani[23]	6	N = 16; Endogenous Dep, consecutive sample	TIMN	100-150	Improvement between the 2 groups was not significantly different at the end of week 1 or 2, but it was highly significant at the end of week 4 and the 6	In two cases TIMN was discontinued S/E reported: Flushing, sweating, dryness of mouth, constipation, postural-hypotension
Dua *et al*.[24]	6	N = 18; DBCT, Depressive Sx in schiz RDC, HDRS >17	CPZ + IMN Vs CPZ + PLB	CPZ upto 1200, IMN 75-150	Both the groups showed significant reduction in dep. Sx after 6-week trial and the addition of IMN to CPZ therapy did not have any advantageous or deleterious effect.	IMN treated group did not exhibit more side-effects than the group receiving PLB

DBCT - Double Blind Controlled Trial; AMT - Amitriptyline; PLB - Placebo; S/E - Side-effects; IMN - Imipramine; HDRS - Hamilton Depression Rating Scale; PTP - Protriptyline; TIMN - Trimipramine; schiz- Schizophrenia; RDC - Research Diagnostic Criteria; CPZ - Chlorpromazine

### Active Comparator Group Drug Trials of Efficacy

There have been 18 trials which have compared two antidepressants.[25–41] One trial compared amitriptyline with amitriptyline and trifluperazine combination[42] and one trial compared amineptine vs. amineptine with benzodiazepine.[43] In another trial nortriptyline was compared with nortriptyline plus fluphenazine [Table 3].[44] The duration of these trials have varied from 10 days to five months; however, most of these have been of four to six weeks duration. Sample size has also varied considerably ranging from 20 to 425 and only four trials have included 100 or more subjects. In terms of study design, 10 trials have followed double blind controlled design; five of these also followed adequate randomization. One trial followed single blind randomized control design and another three trials were open randomized controlled trials. Earlier trials used mixed group of depressive subjects, whereas, recent trials have included subjects with major depressive disorder only. All the trials have used standard doses of antidepressants.

**Table 3:** Active comparator group drug trials of efficacy/effectiveness of antidepressants in depressive disorders

Authors	Duration (in weeks)	Sample size/scale/design	Medication(s)	Dose(s) in mg	Outcome	Side-effects
Chatterjee and Jindal[42]	3-5 months	N = 20	AMT Vs AMT + TFP	AMT 150 AMT 150 + TFP	14/20 (70%), showed good response to depressive symptoms, with 8 reporting complete relief of symptoms. Results were better when TFP was added (75% Vs 66)	TFP reduced the S/E of AMT
Neki[25]	12	N = 200	IMN Vs NLD Vs PLZ Vs PLB	IMN 25-200 NLD 25-200 PLZ 15-120	IMN was the most effective of antidepressant among the 3 drugs IMN and NLD better than PLB PLZ not better than PLB	IMN produced more S/E than NLD or PLZ S/E appeared most frequently during week 2 or three of trial.
Teja and Narang[26]	4	N = 30;HDRS, DBRCT	GO 2998 vs GO 2330 vs IMN		77.5% of subjects in IMN group, 55.5 in GO 2998 and 44.4 in GO 2330 groups had more than 50% reduction in HDRS	Maximum S/E were observed with Go 2998 and then with IMN. Go 2330 had the least number of S/Es
Kumar *et al*.[27]	NA	N = 50	NVL vs IMN	NA	NVL appears to be a promising and safe treatment for depression	NVL relatively free of severe S/E Most common S/E were postural hypotension and coarse tremors of hands, dryness of mouth, sinus tachycardia, drug rash
Teja and Bhatia[28]	6	N = 59; HDRS, DBRCT	IPL vs IMN	IPL -180 IMN -150	Out of the 59 subjects, 47 completed the 4 weeks period of trial and 12 dropped out (6 each on IPL and IMN) after taking the drugs for a variable period In the IMN group 2 subjects were rated as recovered, 5 as markedly improved, 5 as improved and 5 as unchanged where as in the IPL group no subject was rated as recovered, 4 were rated as markedly improved, 5 as improved and 5 as unchanged On t test- neither at day 0 nor at days 28 and 42 was any significant difference seen in the mean scores of any of the Hamilton’s scale Sx between the two drug groups.	There was no difference in the frequency of observed clinical side-effects between the two groups of subjects S/E of severe intensity were seen only with IMN
Satija *et al*.[29]	6	N = 40; HDRS, DBCT	TIMN vs IMN	TIMN -150 IMN -150	At 4 weeks, 16 subjects (80%) recovered with TIMN as compared with 14 subjects (70%) with IMN. There was no effect in 4 subjects with TIMN and 6 subjects with IMN and there was no significant difference between the 2 groups At 6 weeks total number of improved cases remained the same in both group and none of the case worsened	Commonest S/E with TIMN were giddiness, weakness, difficulty in walking, drowsiness and dryness of mouth
Desouza and Chaudhary[30]	4	N = 64; Modified HDRS, DBCT	STL vs IMN	STL -150-250 IMN -150-250	69% of cases treated with STL showed a good response, i.e. reduction in total global score by 50% or more in comparison to only 40.9% of cases treated with IMN responded favorably and this difference was significant In the subgroup analysis it was seen that 90% of subjects in STL showed good response in comparison to only 30% in the IMN group	There were 5 instances of S/E with STL and 13 with IMN In the IMN group, 7 subjects complained of severe S/E and hence the treatment had to be stopped during the first week in 5 and in the third week in 2
Mahal *et al*.[44]	4	N = 100; HDRS, DBCT	FPZ + NTP (20 vs 40) vs NTP (20 vs 40) vs FPZ	FPZ- + NTP- 20-40	Only FPZ group showed 63% reduction in HDRS and NTP 20 mg/day group showed 47% reduction in HDRS with other groups falling between the above 2 groups. However, the difference in mean reduction between the treatment groups is not statistically significant	Headache, dizziness, body pains and burning sensation were commonly complained of by subjects in all the groups. These complaints were more numerous and intense in those who showed poor response to treatment.
Chaturvedi *et al*.[31]	4	N = 30; DBRCT, HDRS	DTP Vs IMN	DTP 25-150 IMN 25-150	Mean percentage reduction in total HDRS score was more with DTP than with IMN throughout the treatment period, though there was no significant difference between the two treatments in terms of overall response and in the response of target Sx	S/E encountered were mild, more in IMN group (dryness of mouth and constipation) than DTP (ataxia and giddiness)
Mahendru *et al*.[32]	4	N = 40; HDRS, ICD-9	NTZ vs DXN		There was more than 75% improvement in total HDRS score in 80% of subjects treated with NTZ as compared to 35% subjects receiving DXN NTZ was found more effective in controlling self reproach and guilt feelings, agitation and social withdrawal	None of the subjects on NTZ reported any unwanted or undesirable effects, while one fourth subjects treated with DXN had side-effects like dryness of mouth, giddiness and drowsiness
Vyas *et al*.[33]	6	N = 60; HDRS, SBRCT	DTP vs IMN	DTP -50-150 IMN 50-150	There was no significant difference between the two groups in terms of reduction in total Hamilton scores	Three subjects receiving DTP and one receiving IMN developed hypomanic/manic features 14/27 subjects in DTP reported side-effects, while 12/23 subjects receiving IMN reported S/E
Agarwal *et al*.[43]	6	N = 40; HDRS, Open trial	AMN vs AMN + BDZ vs BDZ alone	100-200	HDRS scores improved significantly from baseline to the end point (reduced from 22.8 to 11.9) The significant improvement on HDRS was evident as early as 2 weeks 21 out of 37 subjects improved (56.8%) to a moderate degree or more, 21.6% of subjects were reported to have slight improvement whereas 21.6% showed no change or worsening in their conditions Did not present the comparison analysis of the 3 groups	AMN was well tolerated. The S/E reported included dryness of mouth, epigastric pain, constipation, jaundice, headache, insomnia, flushing, and restlessness There was a small but statistically significant increase in body weight No cardiovascular and biochemical A/E were noted
Parikh *et al*.[12]	6	N = 67; DSM-IIIR Dysthymia/MDD HDRS, HARS, MADRS	TPN vs AMT	TPN 37.5- 75 AMT 37.5-75	Both TPN and AMT led to significant improvement in depression from the 2nd week onwards which was sustained till the end of the study(*P* < 0.05)	Subjects on TPN had significantly fewer anticholinergic S/E than those on AMT
Srivastava *et al*.[34]	6	N = 159; HDRS, CGI, DBRCT	CTZ vs IMN	CTZ -40 to 120 IMN -50 to 150	The antidepressant efficacy of CTZ is similar to IMN	Anticholinergic S/E were 4 times less in the CTZ group
Mathur *et al*.[35]	6	N = 39; DSM-IV, HDRS, CGI Open RCT	MTZ vs AMT	MTZ 15-45 AMT 25-150	MTZ is effective in major depression and its efficacy is equivalent to AMT HDRS reduced by 89.91% in the MTZ group and by 54.04% in the AMT group	MTZ was better tolerated than AMT Only six subjects (28.57%) reported S/Es, in contrast to 17 (94.45%) subjects in the AMT group
Vaya *et al*.[36]	4	N = 214; DBRCT multicentric ICD-10, HDRS, CGI	CPM vs ESC vs STN	CPM 20-40 ESC10-20 STN 50-150	Response rate (50% reduction in HDRS) at the end of 4 weeks was 90% for ESC, 86% for CPM and 97% for STN Remission rate (HDRS < 8) at the end of 4 weeks was 74% for ESC, 65% for CPM and 77% for STN	45% of subjects in ESC group, 58% subjects in CPM and 56% subjects in the STN group reported A/E
Avasthi *et al*.[37]	6	N = 60; HDRS, MADRS, open RCT, ICD-10	MCB vs IMN	MCB300- 600 IMN 75-300	IMN better than MCB 62% of subjects in MCB group and 84% in the IMN group were classified as responders on HDRS There were 52.38% responders in the MCB group and 73.68% in the IMN group on MADRS	Subjects who received MCB had a better S/E profile
Mathur *et al*.[38]	6	N = 40; DSM-IV HDRS, CGI, Open RCT	CPM vs AMT	CPM 20-60 AMT 75-150	The percentage reduction in the mean HDRS score for the CPM group was 72.12%, while that for the AMT group was 67.93% and there was no statistical difference between the two groups	20% of subjects in the CPM group reported S/E whereas 75% of subjects in AMT group reported S/
Badyal *et al*.[39]	6	N = 26; MDD, DSM-IV, open RCT, HDRS, MADRS, CGI	DLT vs VFN	DLT 20-40 VFN 75-150	There was significant reduction in HDRS, MADRS, CGI scores from baseline to endpoint (*P* < 0.05) in both the groups There was no significant difference between two groups Response and remission rate was 96% and 69% in DLT group as compared to 92% and 62% in VFN group respectively	There was no significant difference in A/E and laboratory investigation in two groups
Matreja *et al*.[40]	6	N = 100; ROT/ HDRS, MADRS, ADI	CPM vs STN	CPM 20-60 STN 50 150	Significant improvement was seen in HDRS, MADRS, ADI (*P* < 0.05) Decrease in score was more with CPM (*P* < 0.05) Onset of action of CPM was earlier as compared to STN (*P* < 0.05). The number of responders and remitters was also more with CPM (*P* < 0.05)	No serious A/E was reported in either of the groups
Dube *et al*.[41]	8	N = 425 (of which 363 from India) multicentric DBRCT, MDD, DSM-IV, HDRS, QIDS-SR	LY2216684 vs ESC vs PLB		LY2216684 did not show statistically significant improvement from baseline compared to PLB on HDRS ESC demonstrated statistically significant improvement compared to placebo on the HDRS total score at week 8 only (1-sided *P* = 0.057) using the a priori criteria for assay sensitivity of *P* < 0.18 No difference between LY2216684 and PLB on CGI Both LY2216684 and ESC showed significant improvement on QIDS-SR compared to PLB	Subjects treated with LY2216684 exhibited no significant worsening in suicidal ideation as assessed by BSI. No statistically significant differences were observed between groups in rates of discontinuation due to A/E (4 subjects (1.5%) in the LY2216684 group, one patient (0.7%) in the PLB group, and one patient (1.6%) in the ESC group). No deaths were reported during the study. No difference in S/E profile between LY2216684 and ESC, except for higher rate of eosinophilia in LY2216684 group.

AMT - Amitriptyline; TFP - Trifluperazine; S/E - Side-effects; IMN - Imipramine; NLD - Nialmide; PLZ - Phenelzine; PLB - Placabo; HDRS - Hamilton Depression Rating Scale; DBRCT - Double Blind Randomized Controlled trial; NVL - Noveril; IPL - Iprindole; DBCT - Double Blind controlled trial; TIMN - Trimipramine; STL - Sintamil; FPZ - Fluphenazine; NTP - Nortriptyline; DTP - Dothiepin; NTZ - Nitroxazepine; DXN - Doxepin; SBRCT - Single Blind Randomized Controlled Trial; AMN - Amineptine; BDZ - Benzodiazepine; MDD - Major Depressive Disorder; HARS - Hamilton Anxiety Rating Scale; MADRS - Montgomery Asberg Depression Rating scale; TPN - Tianeptine; FLX - Fluoxetine; CGI - Clinical Global Improvement scale; CTZ - Centpropazine; MTZ - Mirtazapine; CPM - Citalopram; MCB - Moclobemide; RCT - Randomized Controlled Trial; DLT - Duloxetine; VFN - Venlafaxine; ROT - Randomized Open Trial; ADI - Amritsar Depression Inventory; STN - Sertraline; QIDS-SR - Self-rated Quick Inventory of Depressive Symptomatology; ESC - Escitalopram; BSI - Beck’s Scale for Suicidal Ideation

Of the 21 trials, 18 have assessed the outcome of depression at the end of trial on Hamilton depression rating scale. Findings from these trials suggest that imipramine is superior to Nialmide,[25] phenelzine,[25] Go 2998,[24] Go 2330[26] and moclobemide[37] in the treatment of depression. Antidepressants like noveril,[27] iprindole,[28] trimipramine,[29] dothiepin[31] and centpropazine[34] have efficacy similar to imipramine. Imipramine has been found to be inferior to sintamil.[30] The study which evaluated amitriptyline and trifluperazine combination showed that it was no better than amitriptyline alone.[42] Interestingly, the study which used fluphenazine found it to be as efficacious as nortriptyline.[44] Nitroxazepine has been shown to be better than doxepin in treatment of depression.[32] The amineptine trial, didn’t present data with regard to comparison in efficacy between the various groups of medications.[43] The studies which have compared various selective serotonin reuptake inhibitors have shown that these are equally effective, except for one which showed that citalopram was better than sertraline.[41] The only trial done on mirtazapine suggests that it is better than amitriptyline.[35] Studies have also shown that citalopram[38] and tianeptine[11] are as efficacious as amitriptyline. The trial by Badyal *et al*.[39] suggests that duloxetine is as efficacious as venlafaxine in the treatment of major depression. One of the recent multicentric trials have shown that escitalopram is superior to investigational drug LY2216684.[41]

Active Comparator Group (non-pharmacological treatment/electroconvulsive therapy) Trials of Efficacy/Effectiveness.

One study has compared the usefulness of antidepressants with respect to non pharmacological treatment[45] and two studies have compared antidepressants with electroconvulsive therapy for treatment of depression.[46,47] Another study compared antidepressants with both electroconvulsive therapy (ECT) and non-pharmacological treatment [Table 4].[48]

**Table 4:** Active comparator group non-drug trial of efficacy of antidepressants in treatment of depressive disorders

Authors	Duration (in weeks)	Sample size/scale/design	Medication(s)	Outcome	Side-effects
Balkrishna *et al*.[45]	2 months	N=75	AMT + CDP vs PPT	Clinically, drug therapy was found to be more effective and economical. However, PPT was effective in relieving the anxiety and depression as well as improving social adjustment. Drug therapy was effective only in the relief of depression.	
Gangadhar *et al*.[46]	12	N = 32; DBRCT, HDRS, Feighner’s criteria, Abrahams criteria for Endogenous dep	IMN vs ECT	Both treatments produced equally significant improvement which was maintained till the end of 6 months. ECT produced its effects quicker.	Subjects who received ECT reported lesser subjective S/Es.
Selvan[47]	4	N = 30; RCT, MDD, DMS-IV, HDRS, MADRS, CGI, BDI	IMN vs ECT	Rates of remission (HRSD < 8) in ECT and IMN group were 71 % and 78% respectively. No significant difference in the two groups on HDRS, MADRS, CGI and BDI.	S/E scores at 2 and 4 weeks were lower in ECT group.
Janakiramaiah *et al*.[48]	3	N = 45; BDI, HDRS	IMN vs ECT vs SKY	No significant difference in 3 groups with regard to reductions in the total scores on BDI and HDRS. Remission (HDRS < 7) at the end of the trial - 93%, 73% and 67% in the ECT, IMN and SKY groups, respectively.	No clinically significant S/Es observed.

AMT - Amitriptyline; CDP - Chlordiazepoxide; PPT - Psychophysiological therapy; DBRCT - Double Blind randomized Controlled Trial; HDRS - Hamilton Depression Rating Scale; IMN - Imipramine; ECT - Electro Convulsive Therapy; S/Es - Side-effects; RCT - Randomized Controlled Trial; MDD: Major Depressive Disorder; MADRS - Montgomery Asberg Depression Rating Scale; CGI - Clinical Global Improvement scale; BDI - Beck’s Depression Inventory; SKY - *Sudarshan kriya*; AD - Antidepressants

One of these studies has shown that pharmacotherapy is more effective and more economical than non-pharmacological treatment.[45] However, one study showed no difference between ECT, antidepressant and *Sudarshan kriya* in the management of depression over the period of three weeks.[48] The studies which compared ECT with imipramine didn’t find any difference in efficacy between the two;[46,47] however, Gangadhar *et al*. reported quicker response with ECT compared to imipramine.

### Dosing Studies of Antidepressants [Table 5]

Seven trials have evaluated the different dosing schedules for treatment of depression.[49–56] These studies suggest that parenteral imipramine is better than oral imipramine and possibly the onset of action is also earlier.[49] Studies have evaluated single dosing versus multiple dosing have shown no difference in efficacy[50–52,54,55] except for one study, which showed that single dose nitroxazepine was better than divided doses.[53]

**Table 5:** Dosing studies of antidepressants

Authors	Duration (in weeks)	Sample size/scale/design	Medication(s)	Dose (s) in mg	Outcome	Side-effects
Chatterjee and Dayal[49]	6-12	N = 80	IMN IM injection vs oral	25 -100	Parenteral IMN more effective Response to injections is quicker and the S/E are less evident and greater margin of safety is obtained parenterally	Most common S/E: Dryness of the mouth, severe constipation and tremors
DeSouza *et al*.[50]	2 weeks	N = 3; HDRS	Parental NTZ (2 dosing schedules)	2 dosing schedules (Schedule A- 75 mg- im Schedule B 50 mg im and 50 mg oral/day)	72.72% of subjects receiving “Schedule- A” were rated as recovered by the day 12 and 100% of those in Schedule ‘ B ’ were rated as improved by the 8^th^ day Sx response similar with both dosing schedules	Intramuscular NTZ was well tolerated S/E reported: Giddiness, restlessness, palpitation, breathlessness, chest pain, visual black out, perspiration S/E more commonly seen in doing a schedule
Sharma and Nandkumar[51]	4 weeks	N = 20	DTP (OD dose vs divided doses)	75-150	2 subjects receiving divided doses showed marked improvement, 4 showed moderate improvement, 2 showed slight improvement and 1had no change in his depression 1 subject receiving single dose showed marked improvement, 3 had moderate improvement, 1 had slight improvement, 1 had no change and 3 showed worsening of depression	S/E similar with both the doses
Shah *et al*.[52]	6 weeks	N = 28; HDRS, DBCT	DTP (Single dose vs. divided doses)	225	64.28% in the single dose and 71.42% in the divided dose group had >50% reduction in HDRS No difference in response rate between single and multiple dosing	Dryness of mouth, tremors, burning sensation in the body and constipation were the only side-effects S/E more common with single dose
Singh *et al*.[53]	4-6	N = 57 DBRCT, HDRS	NTZ (single vs. tid dose)	75-150	At both 4 and 6 weeks, OD dose better than divided doses 70% reduction in HDRS score with no significant difference between the 2 dosing schedule at 4 weeks	Common S/E reported were dryness of mouth and constipation No significant difference in S/E profile between single and divided doses
Sharma and Hegde[54]	4 weeks	N = 43; HDRS, DBCT (Baseline HDRS >30)	DXN (Single vs. tid dose)	75-150	94% in the OD dose and 81% in the divided dose group had >50% reduction in HDRS At least 35.29% in the OD dose and 24% in the divided dose group achieved remission No difference in response rate between OD and multiple dosing	S/Es reported were mild and did not require discontinuation of medication Subjects receiving OD dose didn’t report S/E like palpitation, nausea, vomiting or blurred vision
Sharma[55]	4 weeks	N = 30; HDRS, DBCT, Mean Baseline HDRS >35	DTP (Single vs. tid dose)	75	No significant difference between the groups in terms of reduction in mean Hamilton scores Pattern of reduction in the mean Hamilton scores is similar to both the groups	No difference in S/E reported between the 2 dosing schedules
Malhotra and Santosh[56]		N = 16; DSM - IIIR DBRCT	IMN Loading dose vs. conventional dosing		IMN hydrochloride can relieve depression almost completely within 72 hrs, if given in high bolus doses	

IMN - Imipramine; IM - Intra muscular; S/E - Side-effects; HDRS - Hamilton depression rating scale; NTZ - Nitroxazepine; DTP - Dothiepin; DBCT - Double blind controlled trial; DXN - Doxepin

### Prescription Patterns of Antidepressants in Depression

Chakrabarti and Kulhara[57,58] evaluated the antidepressant prescription pattern in a tertiary care hospital for management of depression during acute and continuation phase. For the evaluation of prescription pattern during the acute phase, case notes of 108 cases fulfilling the ICD-10 criteria of depression or recurrent depression (F32 and F33) were examined. Imipramine was the most commonly prescribed antidepressants followed by Fluoxetine. The authors also observed that pharmacotherapy was often deficient in several areas such as, starting doses, rate of increase in dose, maximum doses used, dose titrations, duration of treatment, change of drugs, recording of side-effects and compliance etc. Results regarding norms for adequate doses and periods of treatment before switching drugs, for the kind of subjects included in this study, were unclear. Regarding the continuation phase treatment, the authors observed that it was deficient in about a third (n = 24; 34 %) of the cases, on either of the two parameters i.e., dose of drugs or duration of treatment and the outcome was poorer in those treated inadequately.

### Efficacy/effectiveness in Disorders Other Than Depression Obsessive compulsive disorder/symptoms [Table 6]

One double blind controlled trial has evaluated the efficacy of clomipramine in the treatment of OCD and showed that clomipramine was superior to placebo in the management of OCD.[59] This study also showed that male subjects showed better response than female subjects. Another study evaluated the efficacy of clomipramine in late onset OCD with comorbid Parkinsonism and showed that clomipramine can be used in elderly subjects and in the presence of Parkinsonism.[60] A small open label study evaluated the usefulness of neuroleptic and fluoxetine combination for treatment of obsessive compulsive (OC) symptoms occurring during the course of schizophrenia and showed that addition of fluoxetine leads to significant improvement in OC symptoms.[61]

**Table 6:** Efficacy/effectiveness/usefulness of antidepressants in other disorders

Authors	Duration (in weeks)	Sample size/scale/design	Medication(s)	Dose (s) in mg	Outcome	Side-effects
Ananth *et al*.[59]	12	N = 27 YBOCS/DBCT	CLN Vs PLB	250	At termination, statistically significant improvement (52.8%) in YBOCS score in the drug group and 10 % in the PLB group Improvement in the CLN group at 4, 8 and 12 weeks of treatment and the difference between the two groups was statistically significant Male subjects improved better than the females (78% vs. 51%), and the difference was statistically significant.	Statistically significant difference in the mean prolactin level between PLB and CLN at the end of 4 and 8 weeks but not at 12 weeks Dosage of medication insignificantly correlated with prolactin level at the end of 4, 8 and 12 weeks
Agarwal and Agarwal[61]	12	N = 7/YBOC, PANSS, CGI, Open trial (trt of OC Sx in Schiz)	Neuroleptic + FLX	FLX 40-80	Five subjects showed response in both OC and psychotic symptoms, 2 showed no response At 12 weeks, there was a significant improvement on YBOCS Total scores on PANSS also showed a significant reduction, it was significant on all the three subscales of PANSS Significant reduction in CGI severity scores On CGI 1 patient showed very much improvement, 3 - much improvement, 1- minimal improvement, 2 - no change	2 subjects had mild anorexia another developed moderate anorexia which gradually decreased after four weeks None had worsening of psychotic Sx
Agarwal *et al*.[60]	32	N = 10; Late onset OCD (.40 years) with Parkinsonism; YBOCS	CLN	37.5-75	5 out of 10 subjects showed 100% improvement, 3 showed 75% improvement, 1 showed 50% improvement and 1 showed 40% improvement in YOBCS	
Vyas and Purohit[62]	10 days	N = 61, Insomnia	TIMN vs NZM	TIMN-50 NZM-10	74% subjects in TIMN group and 63% subjects in NZM group responded favourably and difference between the 2 groups was not statistically significant. Subjects displaying depression and anxiety responded better to TIMN	Dryness of mouth seen only in TIMN group
Singh *et al*.[63]	4	N = 90 (GAD, MAD, Dysthymia) HDRS, HARS	IMN vs DPM	IMN 75-150 DPM 15-30	IMN and DPM were found to be equally effective (62.8% vs 62.2%) in reducing anxiety in all subjects. IMN was significantly better than DPM in reducing the level of depression in the depressed group but as effective as DPM in the other two groups. IMN was significantly better for the symptoms of ‘depressed mood’ and ‘retardation’, while DPM was better for Sx of ‘fears’.	Not mentioned
Dua *et al*.[64]	6	N = 18; DBCT; Dep. Sx in Schiz. RDC; HDRS .17	CPZ + IMN vs CPZ + PLB	CPZ upto 1200 IMN 75-150	Both the groups showed significant improvement after the 6-week trial and the addition of IMN to CPZ therapy did not have any advantageous or deleterious effect	IMN treated group did not exhibit more S/E than the group receiving PLB
Pereira and Patel[65]	6	N = 61; CMD, DBRCT	FLX vs IMN	FLX-20 IMN-75 IMN-150	Treatment completion rates were higher with FLX and least with the IMN 150 mg/day	Anticholinergic SEs and giddiness were common in the IMN groups; headaches and restlessness were common in the FLX group
Patel *et al*.[66]	12 months (treatment for 6 months)	N = 450; DBRCT, CMD	FLX vs PLB vs Psycho social treatment (6 sessions)		Psychiatric outcome was significantly better with FLX than with PLB at 2 months, but not over the 2-12 month period AD were significantly more cost effective than PLB in the short term and long term (*P* < 0.05) Psychological treatment was not more effective than PLB for any outcome during either period	
Dhikav *et al*.[67]	8	N = 68; Open label, PME	FLX vs Yoga		All 38 subjects (25-65.7% 5 good, 13- 34.2% 5 fair) belonging to yoga and 25 out of 30 of the FLX group (82.3%) showed statistically significant improvement in PME	Commonly reported S/E of FLX- nausea, insomnia, vomiting, anxiety

YOBCS - Yale - Brown Obsessive Compulsive Scale; DBCT - Double Blind Controlled Trial; CLN - Clomipramine; PLB - Placabo; PANSS - Positive and Negative Syndrome Scale; CGI - Clinical Global Improvement Scale; OC - Obsessive Compulsive; schiz - Schizophrenia; OCD - Obsessive compulsive disorder; FLX - Fluoxetine; TIMN - Trimipramine; NZM - Nitrazepam; GAD - Generalized anxiety disorder; MAD - Mixed anxiety and depression; HDRS - Hamilton Depression Rating Scale; HARS - Hamilton Anxiety Rating Scale; IMN - Imipramine; DPM - Diazepam; DBCT - Double Blind Controlled Trial; Schiz - Schizophrenia; RDC - Research Diagnostic Criteria; CPZ - Chlorpromazine; CMD - Common Mental Disorders; PME - Premature ejaculation

### Insomnia [Table 6]

One study evaluated the efficacy of antidepressants in insomnia and showed that trimipramine was similar to nitrazepam for treatment of insomnia, especially in the presence of anxiety and depression; however, it had poor tolerability as compared to nitrazepam.[62]

### Generalized Anxiety Disorder [Table 6]

One trial included subjects with generalized anxiety disorder, mixed anxiety depression and dysthymia and showed that imipramine was as effective as diazepam for anxiety symptoms and better than diazepam for the depressive symptoms.[63]

### Depressive Symptoms in Schizophrenia [Table 6]

One trial used imipramine in combination of chlorpromazine and compared it with chlorpromazine alone in the treatment of depressive symptoms in schizophrenia and didn’t find any benefit of adding imipramine to chlorpromazine in the treatment of treatment of depressive symptoms in schizophrenia.[64]

### Common Mental Disorders [Table 6]

Two studies have also studied the usefulness of antidepressants in common mental disorders. One study showed that treatment completion rates were higher with fluoxetine than imipramine.[65] The trial by Patel *et al*.[66] included subjects with common mental disorders and evaluated the outcome at one year. It can be considered the longest study which has evaluated the effectiveness of antidepressant in Indian subjects.

### Sexual Dysfunction [Table 6]

Various sexual side-effects of antidepressants have been utilized for the management of sexual dysfunction. In a recently published open trial Dhikav et al.[67] compared fluoxetine with yoga for the management of premature ejaculation. The study included 68 subjects, of whom 38 were in the yoga group and 30 subjects in the fluoxetine group. All 38 subjects (25-65.7% 5 good, 13-34.2% 5 fair) of yoga group and 25 out of 30 of the fluoxetine group (82.3%) had statistically significant improvement in premature ejaculation and the difference between the two groups was statistically significant too. In an open clinical study, Prusty and Rath (2000)[68] found clomipramine effective in nocturnal emission. In another open trial, Prusty *et al*. (2003)[69] found clomipramine 5 mg along with Sildenafil 50 mg was successful in preventing premature ejaculation of 18 men who had erectile dysfunction also.

### Childhood Onset Disorders [Table 7]

Two studies have evaluated imipramine for management of enuresis in children.[70,71] In one of these trials,[71] in addition to enuresis, children had other behavioral abnormalities too. These studies have shown that imipramine is useful for management of enuresis and also for behavioral problems like obstinacy and temper tantrums. It was further seen that compared to subjects with mental retardation, the response to imipramine was better in children with average intelligence.

**Table 7:** Studies evaluating the efficacy of imipramine in childhood onset disorders

Authors	Duration (in weeks)	Sample size/scale/design	Dose (s) in mg	Outcome
Chatterjee and Khandpur[70]	6-8	N=22 open label, (5-14 years) enuresis	25-100	Good response (<1 week) - 4 cases Delayed response (2-4 weeks) -14 cases No response - 2 cases Abandoned - 2 (1-S/E; 1- otherwise)
Mahendru *et al*. 1970[71]	12	N=75 open label, behaviorally disturbed children age - 4 to 15 years	20-50	58 of the 75 completed the trial At 4 weeks - 46% of enuretic subjects recovered completely, 21% partially and 32% had no improvement At 12 weeks - complete recovery - 68%; partial - 8%; no recovery - 24% Recovery more in subjects with mild enuresis at the beginning At 12 weeks - in obstinacy and temper - 53% favorable response, 47% - no improvement Better response in intellectually average group (73%); compared to mentally retarded children (52%)

### Usefulness in other conditions [Table 8]

Besides the above studies, case series and case reports have also shown usefulness of antidepressants in the management of trichotillomania with trichobezoar,[72] Atypical bulimia Nervosa,[73] Skin Picking,[74] Persistent developmental stuttering,[75] primary hypersomnia,[76] cognitive functioning in depression,[77] proctalgia fugax with dysthymia,[78] palmar-plantar hyperhidrosis,[79] severe resistant depression[80] etc. One open label study also evaluated the usefulness of sertraline in chronic tension type headache and showed that sertraline leads to significant reduction in mean analgesic intake per week, but there is no difference in reduction of headache index and frequency of headache.[81]

**Table 8:** Usefulness of antidepressants (findings from case reports/case series/descriptive studies)

Antidepressant	Condition
Fluoxetine	Trichotillomania with trichobezoar[72]
	Atypical bulimia Nervosa[73]
	Skin Picking[74]
	Persistent developmental stuttering[75]
	Primary hypersomnia[76]
	Improves cognitive functioning in depression[77]
Dothiepin	Proctalgia fugax with dysthymia[78]
Paroxetine	Palmar-plantar hyperhidrosis[79]
Tranylcypromine	Severe resistant depression[80]
Zemelidine	Obsessive Compulsive disorder[81]
Sertraline	Chronic tension type headache:[82] Sertraline led to significant reduction (*P* > 0.05) in mean analgesic intake per week, however, there was no difference in reduction of headache index and percentage reduction in frequency of headache was not significant in sertraline group compared to placebo
	Late onset pedophilia[83]
Escitalopram	Transvestic fetishism[84]
Fluoxetine, Sertraline	Trichotillomania in children and adolescent[85]
Amitriptyline	Clozapine induced sialorrhea[86]

### Tolerability of Antidepressants

As is evident from Tables 1 to 6, tricyclic antidepressants are poorly tolerated compared to the newer antidepressants. Additionally, there are multiple case reports implicating various antidepressants for induction of hypomania/mania,[87–96] hyponatremia/Syndrome of inappropriate antidiuretic hormone secretion (SIADH),[97–99] extrapyramidal symptoms,[100] acute colonic (pseudo) obstruction (Ogilvie syndrome),[101,102] psychosis,[103] hypertension,[104] vascular headache,[105] torsades de pointes,[106] alopecia,[107] cardiogenic shock,[108] seizures,[109,110] galactorrhoea,[111] mania on withdrawal of antidepressants,[112] upper gastrointestinal bleeding,[113] bleeding gums,[114] serotonin syndrome[116,117] etc [Table 9].

**Table 9:** Side-effects of antidepressants

Side-effects	Antidepressant implicated
Antidepressant Induced/associated hypomania/mania	Venlafaxine 150 mg/day[87,96] Escitalopram 20 mg/day[88] Clomipramine 150 mg/day[89,90] Tricyclic induced mania[91] Fluoxetine 60 mg/day[92] Sertraline 50 mg/day[93,94] Citalopram[95]
Antidepressant associated hyponatremia/SIADH	Citalopram 10 mg/day in elderly subjects[97] Escitalopram 10-15 mg/day in elderly subjects[98] Sertraline[99]
Antidepressant associated extrapyramidal Symptoms	Fluoxetine induced akathisia 20mg/day[100]
Acute colonic (pseudo) obstruction (Ogilvie syndrome)	Fluoxetine 80 mg/day and amitriptyline 150 mg/day[101] Clomipramine 50 mg/day[101] Imipramine 150 mg/day (on withdrawal)[102]
Antidepressant Induced/ associated psychosis	Bupropion 300 mg/day[103]
Hypertension	Venlafaxine 150 mg/day[104]
Vascular Headache	Fluoxetine 20 mg/day[105]
Torsades de pointes	Dothiepin 25 mg/day[106]
Drug induced alopecia	Fluoxetine 40 mg/day[107]
Cardiogenic Shock	Imipramine 150 mg/day[108]
Seizures	Fluoxetine 20 mg/day[109] Mirtazapine[110]
Galactorrhea	Paroxetine 25 mg/day[111]
Mania due to antidepressant withdrawal	Imipramine 150 mg/day[112]
Upper Gastrointestinal Bleeding	Sertraline 100 mg/day[113]
Bleeding gums	Duloxetine 40 mg/day[114]
Safety in overdose	Paroxetine 560 mg/day[115]
Serotonin syndrome	Sertraline, trazodone and tramadol[116]
	Lithium carbonate, amlodipine, phenytoin, sertraline, trazodone and escitalopram[117]
Facial Paresthesia/facial	Sertraline[118]
numbness and dysmorphic symptoms	Fluoxetine 20 mg/day[119]
Behavioral activiation and Suicidality	Escitalopram[120]

### Antidepressant Withdrawal/Dependence

One case report presented tricyclic withdrawal syndrome with amitriptyline 300 mg/day[121] and another was described by Jhirwal and Chakrabarti[122] with Venlafaxine. Dependence syndrome has been described with dothiepin 450 mg/day.[123]

### Safety in Overdose

In a case report, Gupta *et al*.[115] described a patient who could tolerate paroxetine 560 mg/day.

### Conclusion and Future Directions

Many studies have evaluated the efficacy of antidepressants in depression and have shown that most of the currently marketed antidepressants are useful. In addition, studies also suggest usefulness of antidepressants in generalized anxiety disorder, dysthymia and common mental disorders. Many of the recent studies have been of good design and have followed double blind randomized controlled design and had reasonable sample size. Further, several studies have been carried out at multiple sites throughout the country. The available data also suggest that antidepressants are more cost-effective than other modalities of treatment for depression.

In addition, there is some evidence to suggest the usefulness of clomipramine in OCD and that of fluoxetine in management of OC symptoms in schizophrenia. However, some major limitations of the research have been that almost all the data available in relation to treatment of depression pertains to acute phase treatment and rarely studies have evaluated the continuation phase treatment. There is also lack of data with regard to the efficacy and effectiveness in the maintenance phase treatment. Surprisingly, no study has evaluated the efficacy/effectiveness of SSRIs in the management of OCD.

There is a need to conduct studies to evaluate the usefulness of antidepressants in the management of panic disorder and depression in medically ill subjects. Studies are also required to evaluate the efficacy of SSRIs in the management of OCD, and to study the usefulness of polypharmacy in the management of depression and other disorders. Studies are few and sparse and there is a need for multi-centric studies in such a vast country.
